# Possible Causes of Extreme Variation of Benzo[a]pyrene Acute Toxicity Test on *Daphnia magna*

**DOI:** 10.3390/toxics12100714

**Published:** 2024-09-30

**Authors:** Zi-Yi Zheng, Yu-Ting Yang, Jing-Xuan Zhou, Zhao-Xing Peng, Hong-Gang Ni

**Affiliations:** School of Urban Planning and Design, Peking University Shenzhen Graduate School, Shenzhen 518055, China; zhengzyi@stu.pku.edu.cn (Z.-Y.Z.); 2001212713@stu.pku.edu.cn (Y.-T.Y.); zhjx@stu.pku.edu.cn (J.-X.Z.); star_pzx@stu.pku.edu.cn (Z.-X.P.)

**Keywords:** benzo[a]pyrene, *Daphnia magna*, toxicity endpoint, acute toxicity

## Abstract

There are enormous differences in benzo[a]pyrene (BaP) acute toxicity tests on *Daphnia magna*, according to previous publications. The explanations of the reasons for this extreme variation are necessary. In this context, the acute toxicity tests of different experiment conditions (light/dark, culture medium, and solvent) were conducted on *Daphnia magna* with BaP as the toxicant of concern. Based on the experiments above, molecular dynamics (MD) simulations were employed to investigate the mechanisms of action. According to our results, the significant influence of light exposure on the acute toxicity test of BaP (*p* < 0.05) on *D. magna* was recorded. On the basis of the MD simulations, it was possible that BaP may not affect the normal operation of Superoxide Dismutase and Catalase directly, and it could be quickly transferred from the body through Glutathione S-transferase and Cytochromes P450. Therefore, when exposed to light, the oxidative stress process intensifies, causing damage to *Daphnia magna*. Apparently, the ecotoxicity tests based on inhibition for *D. magna* cannot adequately reflect the toxic effects of BaP.

## 1. Introduction

Benzo[a]pyrene (BaP) is one of the most important members in the polycyclic aromatic hydrocarbons (PAHs) group, which is widely found in the aquatic environment. Due to its teratogenic, mutagenic, and carcinogenic properties on diverse organisms, the toxicity of BaP has raised concerns for human health and ecological safety [1,2,3]. It has been detected in various aquatic environments, including surface water, seawater, groundwater, and drinking water, as well as in sediment samples, as a result of atmospheric deposition and industrial wastewater discharge [4]. It was reported that Bap concentrations in Chinese rivers ranged from 0.01–382.36 ng/L [5].

The mechanism of action (MOA) is the specific means by which chemicals induce effects in organisms. Environmental scientists have documented that BaP damages aquatic organisms such as disrupting the immune system, causing abnormal energy metabolism and osmotic regulation, DNA damage, and apoptosis [6,7,8]. The metabolism of BaP includes phase I and II metabolism [9]. One crucial mechanism through which BaP exerts its effects is its free radical activity, resulting in the induction of oxidative stress within cells [10,11]. The BaP-induced toxic effect primarily occurs via phase I metabolites. Once BaP enters aquatic organisms, the initial metabolic process that occurs is an oxidation–reduction reaction that produces reactive oxygen species (ROS) and toxic intermediate metabolites [12]. BaP triggers oxidative stress by ROS, disrupting the function of damaging lipids, proteins, and DNA [13].

For the pollutants’ mode of action, compounds can be categorized as narcotic or reactive [14]. Narcotic compounds are further divided into baseline and less inert compounds, which particularly cause cellular toxicity through their hydrophobic properties [15]. The toxic mode of action of BaP should be as weak as that of inert compounds according to the modified Verhhar’s scheme in Toxtree software online (http://toxtree.sourceforge.net/), accessed on 10 October 2023. Previous studies have shown that some compounds may share different modes of action among different test species [16]. Therefore, it may not be reasonable to categorize a compound broadly as a single mode of action. To date, research on the toxicity of BaP in invertebrates seems to mainly focus on modes of action rather than MOA.

**Figure 1:**
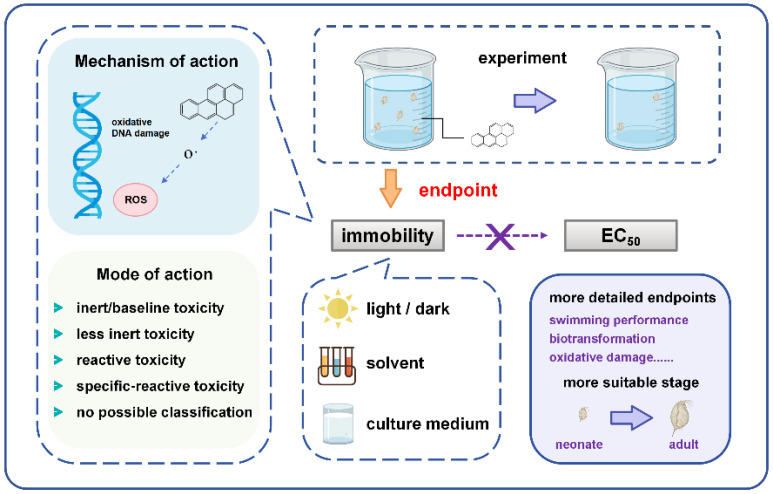
Schematic diagram of proposed research methodology.

There are limited reports on BaP acute toxicity tests on *Daphnia magna*. However, the acute toxicity values in these reports exhibit significant variations. In general, the most frequently reported endpoints for *D. magna* primarily revolve around “immobility”, with little attention given to chronic and sublethal effects such as genotoxicity and reproductive inhibition [17,18]. Apparently, “immobility” may be the most straightforward and primary ecotoxicity endpoint and cannot provide complete information on the MOA of BaP in *D. magna*. Therefore, “immobility” might not be sensitive enough to mirror the ecological risk in aquatic ecosystems. What are the possible causes of the extreme variations in the BaP acute toxicity test on *D. magna*? This study aims to investigate whether relying on inhibition as the primary toxicity endpoint for assessing the toxic effects of BaP is reasonable (Figure 1).

## 2. Materials and Methods

### 2.1. Chemicals

BaP (99%) was purchased from Macklin Inc. (Shanghai, China) and J&K Scientific Co. (Beijing, China). Dimethyl sulfoxide (DMSO) and acetone (AC) were procured from Macklin Inc. (Shanghai, China). Potassium dichromate (K_2_Cr_2_O_7_) used as the reference compound was obtained from J&K Scientific Co. (Beijing, China). BaP was dissolved in DMSO or AC to prepare the stock solution. The total volume of stock solution with AC added was less than 60 μL to ensure that the solvent did not affect the experiment; this value for DMSO was 200 μL.

### 2.2. Test Organism

*D. magna* were obtained from the Laboratory Animal Monitoring Institute of Guangdong Province, China. *D. magna* were cultured in a growth medium at a temperature of 20 ± 2 °C under a 16 h light/8 h dark cycle with an illumination intensity ranging from 1110–1480 lx [19]. *D. magna* were provided daily with *Chlorella* sp. as their food source, which was cultivated in BG11 medium. The International Organization for Standardization (ISO) standard water and dechlorinated tap water (TW) were employed as culture media. The culture medium was renewed three times a week. After three generations of parthenogenesis, under the same conditions, neonatal *D. magna* aged 6–24 h were used for the acute toxicity assessment. In all toxicity tests, the test conditions mirrored the culture conditions. Before the test, a 24 h acute immobilization test with K_2_Cr_2_O_7_ was conducted to assess the sensitivity of neonates. The toxicity test was considered valid when the 24-h EC_50_ of K_2_Cr_2_O_7_ fell within the range of 0.6 to 2.1 mg/L.

### 2.3. Acute Toxicity Tests of BaP

The suitable dilution factor was set as 2 for each test. For the experiments with low concentrations, five concentration levels were selected (0.025 mg/L, 0.05 mg/L, 0.1 mg/L, 0.2 mg/L, and 0.4 mg/L). For the experiments of high concentrations, six concentration levels were selected (0.25 mg/L, 0.5 mg/L, 1 mg/L, 2 mg/L, 4 mg/L, and 8 mg/L). Every experiment was run in triplicate, with 10 neonates (6–24 h old) in a 50 mL conical flask containing 20 mL medium. During the test, no additional food was provided, and the solution was not renewed. To reduce evaporation, all test containers were covered with vented sealing film. After 48 h of exposure, neonates that failed to move within 15 s after gentle agitation of the test vessel were considered to be immobilized. The statistical analysis in this paper was performed using SPSS 27, and the graphs were created using Origin 2022.

### 2.4. Exposure Concentrations

To compare the exposure concentrations to the nominal concentrations, measurements of concentrations of BaP and K_2_Cr_2_O_7_ in the corresponding culture media were conducted. The samples were three consecutively extracted with 20 mL of dichloromethane in an ultrasonic bath. The extracts were combined and spiked with a known number of internal standards and condensed, solvent-exchanged to hexane, and further reduced to 1 mL for gas chromatograph mass spectrometer (Shimadzu, QP2010Ultra, Kyoto, Japan) analysis. A 30 m × 0.25 mm—i.d. (0.25 µm film thickness) DB-5MS column (J&W Scientific, Folsom, CA, USA) was used for separating the target analytes. The column temperature was programmed from 80 °C and maintained for 2 min, then the temperature was increased to 180 °C at a rate of 20 °C/min for 5 min, and finally, the temperature was raised to 290 °C at a rate of 10 °C/min held for 15 min. The concentration of K_2_Cr_2_O_7_ was determined using a spectrophotometer. Recoveries of BaP and K_2_Cr_2_O_7_ were 83.4 ± 9.53% and 99.7 ± 0.83%, respectively. The exposure concentrations were similar to the nominal concentration.

### 2.5. Molecular Docking

The structures of the BaP used for molecular docking were obtained from the PubChem database (http://www.ncbi.nlm.nih.gov/pccompound), accessed on 8 March 2024. The crystal structures of the proteins used in this study (https://alphafold.ebi.ac.uk), accessed on 20 March 2024. The lowest docking score of the combined conformations was regarded as the optimal conformation. Autodock was used for molecular docking calculations, and all water molecules were removed from the structure. Maestro 12.6 was used to draw two-dimensional (2D) images of molecular docking.

### 2.6. MD Simulation

The optimal conformations of the complexes obtained from the molecular docking were utilized in the molecular dynamics (MD) simulation using AMBER22. The force field parameters of ligands, proteins, and water molecules for MD simulations separately stem from the general Amber force field (GAFF2) [20], the Amber ff14SB force field [21] and the TIP3P model [22]. Sodium ions and chloride ions were introduced into the box to maintain the system’s electrical neutrality. In the optimization process, the steepest descent method was applied for 1000 steps, followed by 4000 steps using conjugate gradient minimization. Each system experienced a controlled temperature rise from absolute zero (0 K) to room temperature (298.15 K) over 5 ns. Following equilibration, a 50 ns MD simulation was carried out for every system at 298.15 K and a pressure of 1 atm. After equilibration, a 50 ns MD simulation was conducted for each system at 298.15 K and a pressure of 1 atm. During these simulations, the step length was set to 2 fs, and every frame was recorded as 5000 steps.

## 3. Results and Discussion

### 3.1. Effect of Experiment Conditions

The acute toxicity of BaP for *D. magna* obtained in those previous works ranged from 0.982 μg/L to 250 μg/L, and a three-magnitude difference was observed [23,24,25,26,27]. This enormous difference in toxicity for the same substance on the same model organism deserves further examination. In this context, the actual EC_50_ for BaP in aquatic systems needs reinvestigation under various experimental conditions.

**Figure 2:**
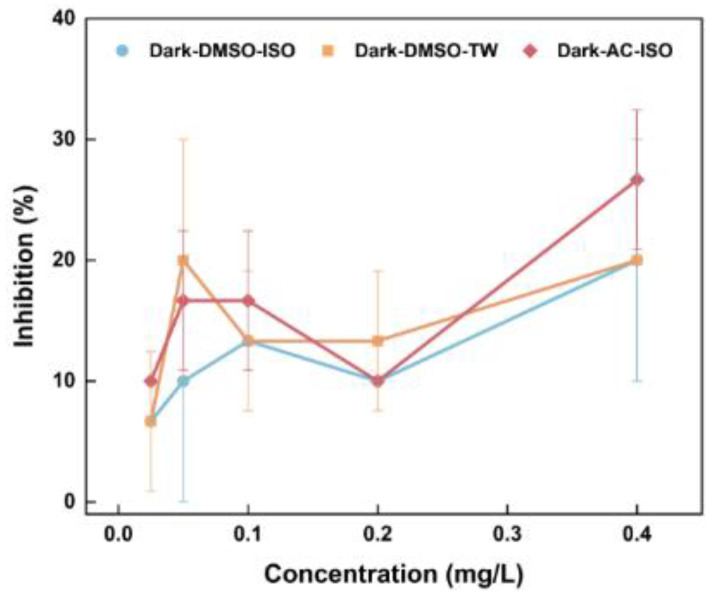
Effects of different solvents and culture media on the toxicity of BaP to *D. magna*.

According to the guidelines of the OECD, light avoidance experiments should be carried out when the test compounds exhibit instability under natural light [19]. Because of the photodegradation of BaP [28,29], our initial experiments were conducted under dark conditions. The acute toxicity tests of BaP with different culture media and solvents are displayed in Figure 2. Within the concentration range of 25 μg/L to 400 μg/L, the inhibition of *D. magna* by BaP is consistently below 30%. No significant differences between the three treatments (DMOS + TW, DMOS + ISO, and AC + TW, all under dark conditions) were observed based on the *t*-test (*p* > 0.05). This result indicates that AC and DMSO as solvents and ISO standard water and dechlorinated tap water as culture media do not have a significant impact on acute toxicity. As higher concentration experiments were conducted next, the presence of AC when adding more BaP could affect *D. magna*, potentially interfering with the experimental assessment. Therefore, considering the severe toxicity of AC to *D. magna*, only DMSO was used as the solvent in subsequent experiments.

### 3.2. Effect of Light

**Figure 3:**
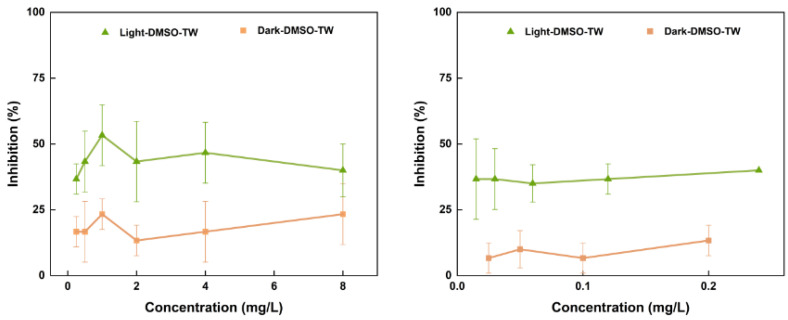
Effects of light and dark conditions on the toxicity of BaP to *D. magna*.

As mentioned above, the EC_50_ cannot be obtained under dark conditions. Again, those previous works did not declare whether light was avoided during testing. Therefore, in this study, experiments with light exposure with higher BaP levels were conducted with the same light duration as the original culture conditions. During the light experiments, only low toxic effects were observed, with BaP levels growing from 25 μg/L to 8000 μg/L (Figure 3). Moreover, under light, regardless of changes in concentrations, the inhibition of *D. magna* always stabilized at ~50%. For both light and dark experiments, there was no obvious trend in the inhibition of BaP on *D. magna* with respect to concentration variations, making it unfeasible to calculate the EC_50_. A *t*-test showed that there was a significant difference between the light and dark experiments (*p* < 0.05), suggesting that light could improve the inhibition of BaP on *D. magna*.

**Figure 4:**
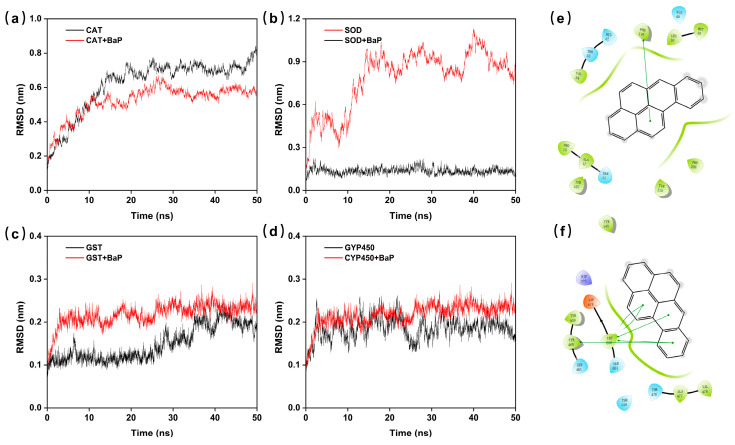
The MD simulation of enzymes and BaP. (**a**–**d**) The RMSD values of the apo state and docked state during the MD simulation. (**e**,**f**) The molecular docking of ligands with the CYP450 and GST protein.

To further explore the possible mechanisms of action, we selected enzymes that significantly influence the oxidative stress process in *D. magna*, i.e., Catalase (CAT), Superoxide Dismutase (SOD), Glutathione S-transferase (GST), and Cytochromes P450 (CYP450), to conduct molecular docking and molecular dynamics (MD) simulations with BaP. A 50 ns MD simulation of the dynamics of four complexes was conducted. The root-mean-square deviation (RMSD) of GST + BaP and CYP450 + BaP did not fluctuate greatly (<0.3 nm), which indicated that there was a stable system and stronger binding between the compound and the protein (Figure 4c,d). As is shown in Figure 4e,f, Pi–Pi stacking interactions (including van der Waals interactions and electrostatic interactions) were formed between proteins and residues, which increased the binding stability. The bindings of BaP with SOD and CAT were poor, suggesting that BaP does not occupy these enzymes (Figure 4a,b). CAT and SOD are primarily responsible for helping the body eliminate ROS, preventing oxidative damage to the body. GST and CYP450 are transferases and metabolic enzymes that seek to excrete pollutants or toxins from the body. Therefore, BaP binds with GST and CYP450 stably but not well with CAT and SOD. This not only ensures that the normal removal of ROS in the body is unaffected but also allows BaP to be quickly excreted by metabolic enzymes and transferases. As a result, it does not cause significant harm to *D. magna*.

According to a previous study, no toxic effects on newt larvae for BaP alone were found, but toxic effects in animals exposed to BaP + daylight were observed [30]. Apparently, light is one of the key factors that initiates oxidative stress [31]. Thus, in the absence of light, the oxidative stress process is slow, and the enzymes in *D. magna* can eliminate and transfer most of the free radicals and harmful substances. However, when exposed to light, the oxidative stress process intensifies, causing damage to *D. magna*.

There is a correlation between carcinogenicity and toxicity [32], while BaP is highly carcinogenic; therefore, BaP is considered a highly toxic pollutant. BaP did not exhibit significant toxic effects under conditions of light avoidance, according to our results. Indeed, weak light surroundings are sometimes present in real aquatic environments (such as shaded areas and deep-water zones). Hence, the aquatic toxicity of BaP may be overestimated.

Furthermore, BaP has different degradation rates under different wavelengths of light, which also affects its toxicity to *D. magna* [33]. However, the previous studies did not precisely specify the lighting conditions during the toxicity experiment, which could be a possible explanation for the enormous disparities in the literature data. Therefore, more detailed consideration of light and dark conditions should be pointed out in future studies on BaP. If experiments are conducted under light conditions, they should follow the OECD lighting requirements and be specifically noted in the research. This approach will reduce the discrepancies in the acute toxicity of BaP.

### 3.3. EC_50_ Value of BaP

Under conditions of light avoidance, oxidative stress is delayed, so under conditions of light exposure, oxidative stress should proceed normally. However, for both light and dark experiments, no appropriate data trends and patterns could be used to calculate the EC_50_ of BaP on *D. magna*.

As mentioned above, the carcinogenicity of BaP has always been a concern, while research on the acute toxicity of BaP is comparatively limited. Out of all the investigated biomarkers for oxidative stress, DNA damage is regarded as a potent differentiating factor for assessing the toxic effects of different contaminants. On the one hand, the effects on DNA often require identification through chronic experiments or more detailed observational experiments. For example, Silva et al. used swimming performance, biotransformation, oxidative damage, energy production, and levels of BaP-type compounds in tissues (eye, digestive land, and muscle) during a 96 h acute bioassay with BaP exposure to evaluate BaP toxicity. The EC_50_ obtained from 48 h acute immobilization tests may be insufficient to fully reflect the impact of BaP on *D. magna*. The inhibition of *D. magna* may not necessarily reflect the impact of oxidative stress. Hence, the experiment did not exhibit a significant concentration-response. Therefore, previous studies on the acute toxicity of *D. magna* may have some blemishes. On the other hand, previous studies revealed that adults exhibited higher transcript levels of DNA repair genes and showed significant induction of these genes upon exposure, while neonates did not [17]. Many transcripts encoding genes related to DNA damage and the oxidative stress response exhibited higher basal transcription levels in adults than in neonates. Compared to neonates, adults are more likely to respond to DNA damage and oxidative stress. Therefore, in toxicity tests of chemicals such as BaP that primarily induce DNA damage through oxidative stress, neonates may not be the most suitable test invertebrate species. These are possible factors that may explain the lack of a trend in BaP acute toxicity, but the specific reasons require further experiments and investigation.

## 4. Conclusions

The acute toxicity of BaP is significantly affected by light exposure. Immobility is not a suitable endpoint for assessing the toxic response of *D. magna* to BaP. Those previous studies were mainly based on direct EC_50_ or LC_50_ values for risk assessment, without considering the influence of specific endpoints and natural conditions on the results, which could be a reason for the significant disparities in the literature data. Our study demonstrates that BaP did not exhibit significant toxic effects under conditions of light avoidance. While the current approach to acute toxicity testing with *D. magna* is essential for emergency risk management, it may not fully reflect the actual environmental situation, potentially leading to an overestimation of BaP’s ecological risk.*D. magna* Apart from that, we found no appropriate data trends and patterns for both light and dark toxicity data. We found possible reasons from published papers: (1) The impact of oxidative stress on aquatic organisms may not be fully reflected by inhibition but can instead be shown by the induction of organ and cell carcinogenesis. This DNA damage may not have an immediate effect on mortality; (2) transcripts related to DNA damage and oxidative stress showed higher basal levels in adults compared to neonates, indicating that adults of *D. magna* are more responsive to these stresses.

## Data Availability

The data that support the findings of this study are available from the corresponding author upon reasonable request.
